# Two Cases of Cervical Epidural Hematoma Presenting With Left-Sided Hemiplegia and Requiring Surgical Drainage

**DOI:** 10.7759/cureus.23915

**Published:** 2022-04-07

**Authors:** Ryo Yamamoto, Masashi Ito, Hiroki Shimuzu, Kenichi Wakabayashi, Hirofumi Oyama

**Affiliations:** 1 Neurosurgery, Nagoya University, Nagoya, JPN; 2 Neurosurgery, Toyohashi Municipal Hospital, Toyohashi, JPN

**Keywords:** stroke mimics, stroke, hypertension, hemiparesis, surgery, paralysis, spinal epidural hematoma

## Abstract

Spinal epidural hematoma is a rare disease that may present as motor paralysis, sensory disturbance, and a sudden radiating pain from the hematoma site. Herein, we report two cases of cervical epidural hematoma diagnosed as left hemiplegia and treated with surgery. Case 1 was a 62-year-old woman who presented to our hospital with the chief complaint of posterior neck pain and left upper and lower limb paralysis. Cervical magnetic resonance imaging (MRI) showed a cervical epidural hematoma at the C4-C6 level. Case 2 was a 67-year-old man who presented to our hospital with a history of hypertension. Both patients had left hemiparesis, numbness in the left upper and lower limbs, and hypersensitivity. They were diagnosed with idiopathic cervical epidural hematoma and underwent emergency surgery (hematoma removal + laminoplasty). In case 1, the paralysis improved immediately after the surgery. In case 2, the paralysis and hypersensitivity improved markedly after the surgery, and the manual muscle testing grade of the left upper and lower limbs improved from 3 to 5 on the second day. Both patients were subsequently discharged home unaided. In cases where the paralysis does not improve, it is important to exclude stroke, diagnose cervical epidural hematoma as soon as possible, and consider surgery aggressively.

## Introduction

Spinal epidural hematoma is a rare disease that may present with motor paralysis, sensory disturbance, and a sudden radiating pain from the hematoma site. It often presents as sudden paraplegia or tetraplegia due to a compressive lesion in the spinal cord, but it can also occur as hemiplegia, requiring careful differentiation from stroke. In recent years, remarkable progress has been made in the treatment of acute stroke, and treatments such as the administration of recombinant tissue plasminogen activator (rt-PA) and percutaneous cerebral thrombectomy are accepted standard of care for early-onset cerebral infarction in the modern world. In contrast, spinal epidural hematoma is a contraindication for the administration of rt-PA, and if not diagnosed properly, it may lead to accidental rt-PA administration and subsequent deterioration of the patient's condition. Herein, we report two cases of cervical epidural hematoma diagnosed as left hemiplegia and treated with surgery.

## Case presentation

Case 1

A 62-year-old woman presented to our hospital with the chief complaint of posterior neck pain and left upper and lower limb paralysis. She had no relevant medical history.

**Figure 1:**
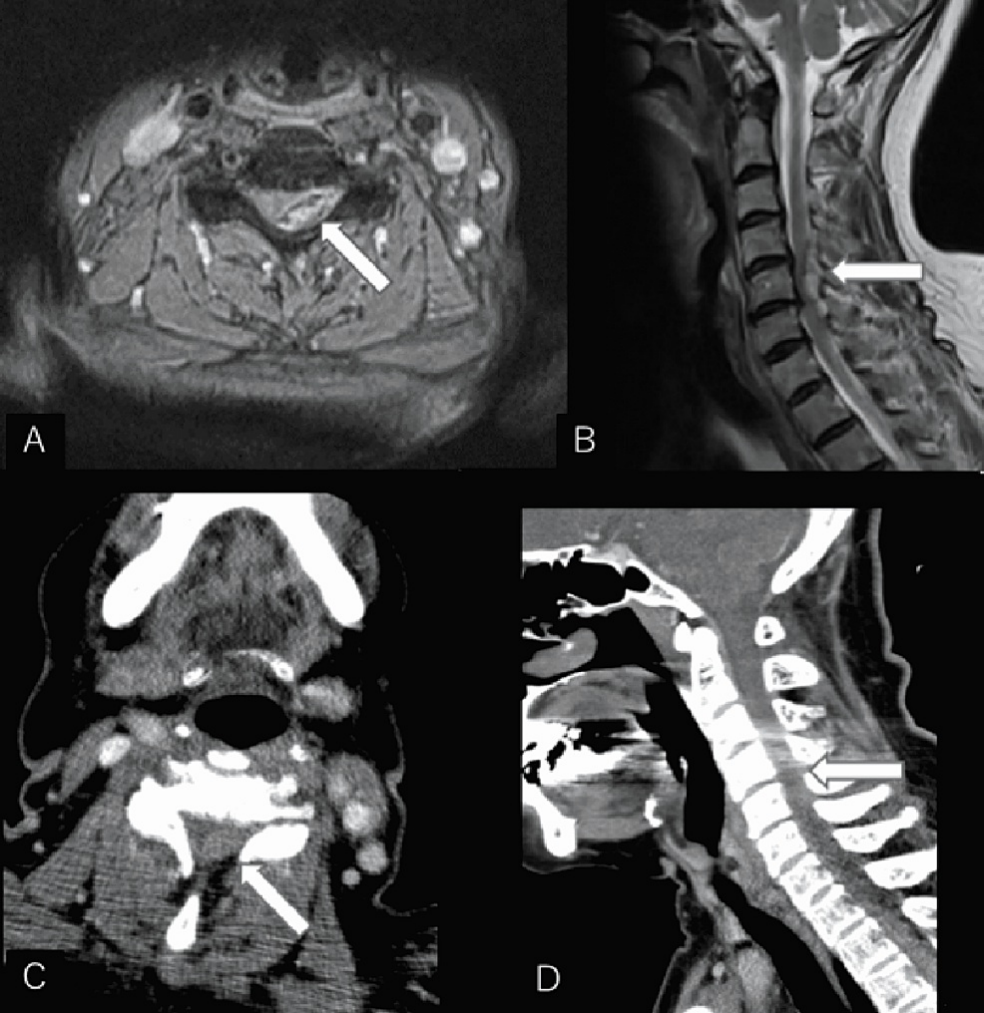
Case 1 preoperative images (A, B) Preoperative cervical T2-weighted MRI images at the C5 level in axial (A) and sagittal (B) views. (C,D) Preoperative cervical CT scan at the C5 level in axial (C) and sagittal (D) views. These show epidural hematoma on the left dorsal side at the C4-C6 level.

The patient experienced a sudden posterior neck pain while cleaning. Two hours later, numbness and incomplete paralysis of the left side of the body occurred, and the patient was referred to a local neurosurgery clinic. A head computed tomography (CT) and magnetic resonance imaging (MRI) were performed, but no cerebral hemorrhage or cerebral infarction was detected. Cervical MRI showed no signs of ligament injury and revealed a cervical epidural hematoma at the C4-C6 level (Figures 1A, 1B), and the patient was referred to our hospital.

Upon arrival, the patient was clearly conscious (Japan Coma Scale 0). She had left hemiparesis (manual muscle testing grade 3, MMT3, in the upper limbs and MMT2 in the lower limbs), numbness in the left upper and lower limbs, and hypersensitivity. There were no other notable neurological findings. The patient’s blood pressure was 202/124 mmHg upon arrival.

**Figure 2:**
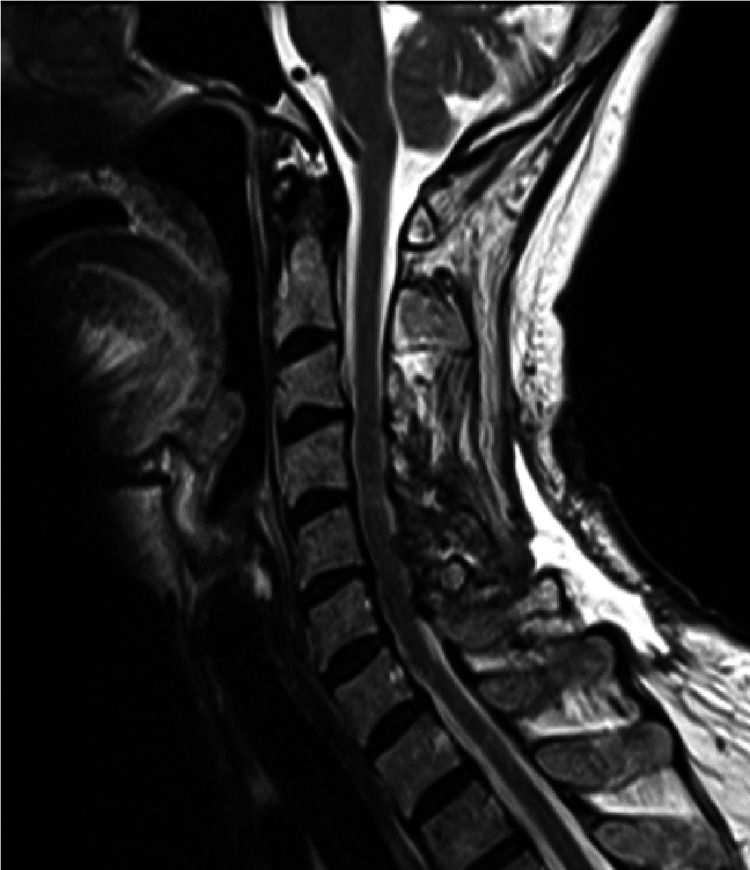
Case 1 postoperative cervical sagittal T2-weighted MRI image

The patient underwent an urgent head and neck contrast CT (Figures 1C, 1D) and cerebral angiography, but there were no obvious vessel abnormalities or wall irregularities. The patient had no liver disease or antithrombotic medications and was diagnosed with idiopathic cervical epidural hematoma at the C4-C6 level and underwent emergency surgery (hematoma removal + C4-C6 laminoplasty). The patient entered the operating room 9 hours after the onset of posterior cervical pain. The paralysis improved immediately after the surgery and disappeared the next day. An MRI scan of the neck was performed on the third day of hospitalization, and the hematoma was completely removed (Figure 2). Cerebral angiography was performed again on the 13th day of hospitalization, but no abnormalities were detected. The patient’s rehabilitation proceeded smoothly, and she was discharged home unaided on the 15th day, with a modified Rankin scale score of 1.

Case 2

A 67-year-old man presented to our hospital with a history of hypertension. The patient was driving a car when he suddenly experienced a posterior neck pain. One hour later, he went to a local doctor, who prescribed painkillers and sent him home. Four hours later, after taking a bath at home, he experienced incomplete paralysis of the left side of his body and was rushed to our hospital.

Upon arrival, the patient’s consciousness was clear (Japan Coma Scale 0). He had left hemiparesis (MMT3), numbness in the left upper and lower limbs, and hypersensitivity. There were no other notable neurological findings. His blood pressure was 219/122 mmHg at the time of admission.

**Figure 3:**
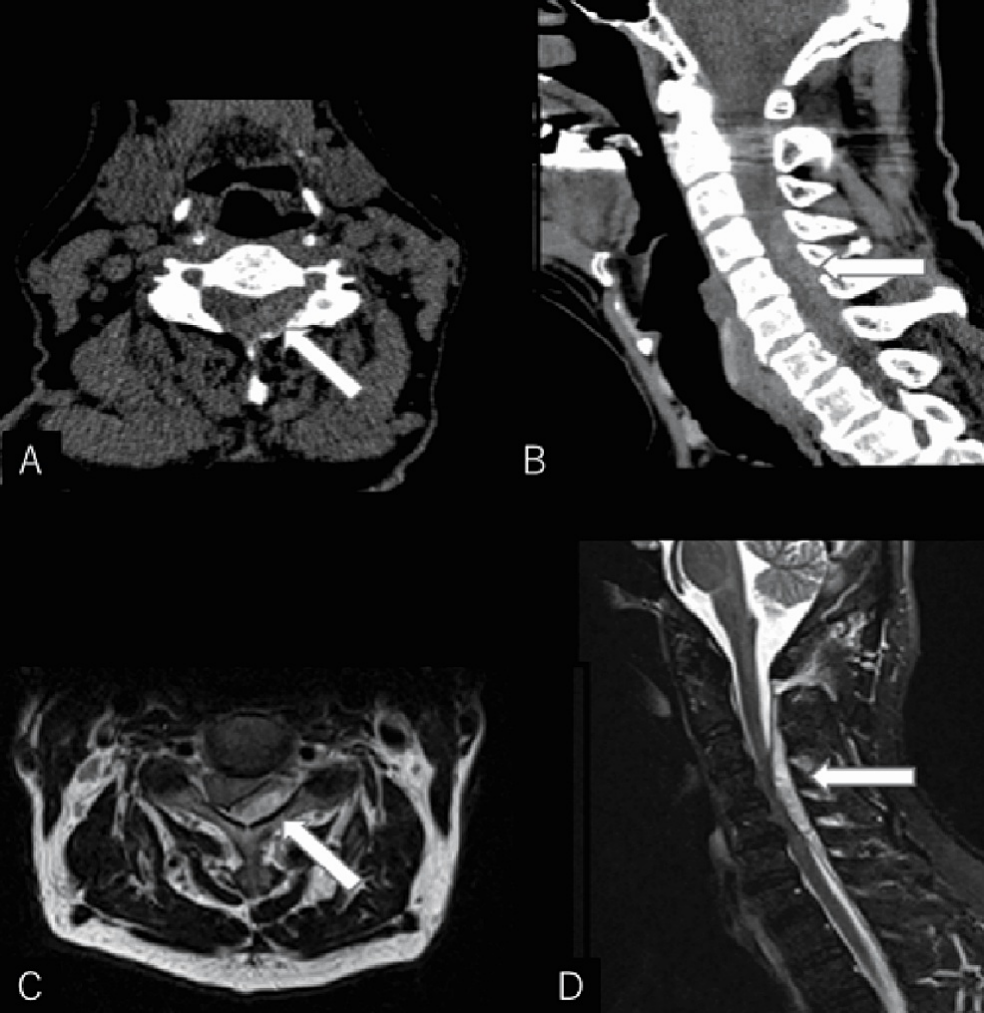
Case 2 preoperative images (A, B) Preoperative cervical CT scan at the C4 level in axial (A) and sagittal (B) views. (C, D) Preoperative cervical T2-weighted MRI images at the C4 level in axial (C) and sagittal (D) views. These show epidural hematoma on the left dorsal side at C3-C6 level.

**Figure 4:**
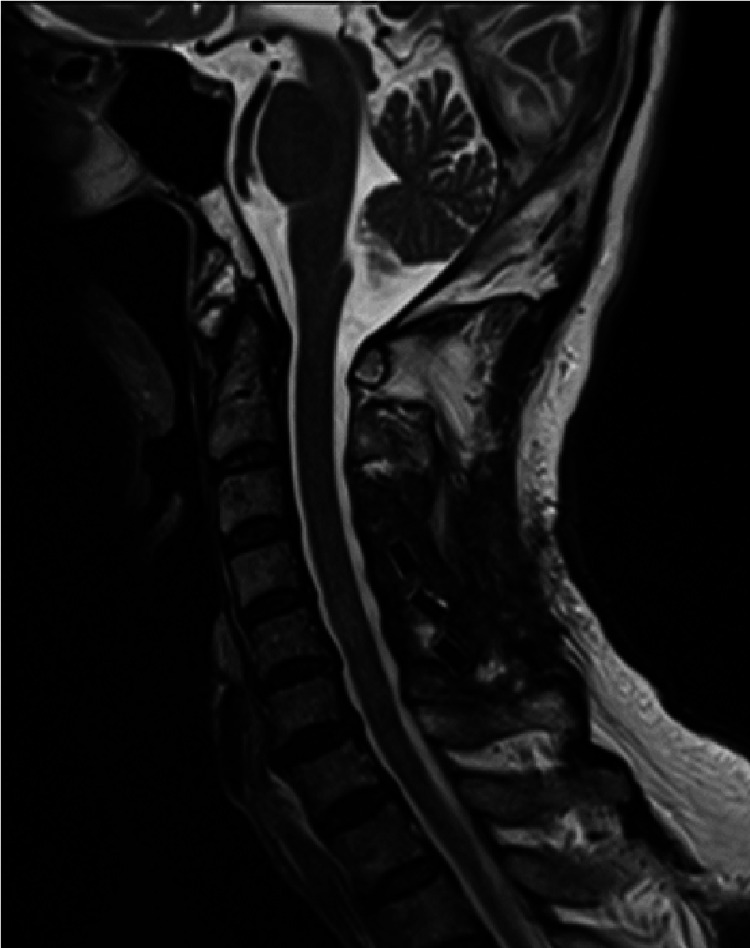
Case 2 postoperative cervical sagittal T2-weighted MRI image

The patient underwent head and neck CT (Figures 3A, 3B) and head MRI, but no intracranial hemorrhage or cerebral infarction could be detected. Spinal epidural hematoma was suspected, and a cervical MRI was performed (Figures 3C, 3D). No abnormal blood vessels were observed. The patient was diagnosed with idiopathic cervical epidural hematoma and underwent emergency surgery (hematoma removal + C3-C6 vertebroplasty). The patient was admitted to the operating room 9 hours after the onset of posterior cervical pain. The paralysis and hypersensitivity improved markedly after the surgery, and the MMT grade of the left upper and lower limbs improved to 5 on the second day. On the third day, a cervical MRI scan was performed, and the hematoma was removed (Figure 4). The patient was discharged home unaided on the same day, with a modified Rankin scale score of 1.

Oral consent was obtained from both the patients for the publication of this case report.

## Discussion

Spinal epidural hematoma is a rare disease with an incidence of 0.1/100,000 per year [1]. Factors associated with the onset of the disease include abnormalities in blood coagulation (antithrombotic medication or bleeding disorders), vascular malformations, pregnancy, and hypertension [2,3]. Groen and Possen analyzed 199 cases from the literature and reported that 24% of the patients had been treated with anticoagulant therapy, 19% had hypertension, 48% had other causes such as trauma or systemic disease, and the remaining 52% had unknown causes [4].

In addition, spinal epidural hematomas are predominantly distributed at the middle and lower cervical spine level (C4-C7), which is thought to be the region of greatest cervical spine mobility [5]. Intracranial epidural hematoma is caused by a disruption of the middle meningeal artery, which is mainly caused by a skull fracture due to head trauma, whereas idiopathic spinal epidural hematoma is caused by the epidural veins around the dural canal, especially the posterior internal vertebral venous plexus, as the source of bleeding without obvious trauma. The epidural venous plexus develops on the lateral side of the spinal canal, and the distribution of the hematoma is likely to be uneven on either side. There have been reports of patients being misdiagnosed as having a cerebral infarction in the emergency room and being treated with acute cerebral infarction therapy such as the administration of rt-PA, resulting in worsening neurological symptoms [6].　

It is important to make an accurate and timely diagnosis of cervical epidural hematoma in order to consider treatment. If a cervical spine epidural hematoma is suspected during a head CT scan for stroke, CT scan of the neck can be performed consecutively, allowing for an early diagnosis. Based on our experience, the characteristic findings of cervical spine epidural hematoma include (1) clear consciousness and complaints of posterior neck pain, back pain, and numbness, (2) the absence of cerebral nerve palsy, and (3) strong sensory hypersensitivity.

Although Horner's syndrome was not observed in any of the cases reported here, it is reported to be a clinical sign that occurs in a high percentage of patients [7]. Therefore, it is important to not confuse symptoms such as unilateral eyelid drooping and contracted pupil with symptoms of cerebral palsy. However, cases without posterior neck pain have also been reported, which may make it more difficult to distinguish from stroke.

Hara et al. reported that hematomas were predominantly found on the left side in 10 cases and on the right side in five cases [7]. In the two cases studied here, the lesions were on the left side, suggesting that there may be some relationship.

The efficacy of surgical treatment and conservative treatment is still controversial. Spontaneous recovery has been reported to be characterized by mild paralysis (MMT3 or higher) and improvement in paralysis within six hours, even in cases of severe paralysis [8]. As mentioned earlier, venous hemorrhage is less likely to increase pressure, and the hematoma can move in the spinal canal, which may support the spontaneous recovery of neurological symptoms. In contrast, the surgical results of patients who have passed more than 6 hours after the onset of symptoms worsen over time, and it is essential to monitor the symptoms regularly [4].

In both of our cases, surgical treatment was not started until about 6 hours after the onset of symptoms, and there was no improvement in paralysis. When symptoms do not improve over time, even with MMT3, surgical treatment is effective and neurological symptoms improve markedly, as in this case; hence, surgical treatment should be considered proactively. Cervical epidural hematoma is an important, albeit infrequent, condition that stroke neurologists, neurosurgeons, orthopedic surgeons, and emergency physicians must keep in mind during their daily practice.

## Conclusions

We encountered two cases of cervical spine epidural hematoma that were treated surgically. Based on our experience, the characteristic findings of cervical spine epidural hematoma include clear consciousness and complaints of posterior neck pain, back pain, and numbness; the absence of cerebral nerve palsy; and strong sensory hypersensitivity. We neurosurgeons need to be well informed about cervical epidural hematoma, and it is important to exclude stroke and diagnose properly and quickly. If the paralysis has not improved by the time the patient arrives and completes the initial consultation, we would suggest that surgery should be aggressively considered.
